# Crystal structure and Hirshfeld surface analysis of 5-(5-phenyl-1,2-oxazol-3-yl)-1,3,4-thia­diazol-2-amine

**DOI:** 10.1107/S2056989022003450

**Published:** 2022-03-31

**Authors:** Evgeniya V. Nikitina, Sevim Türktekin Çelikesir, Mehmet Akkurt, Sergey K. Petkevich, Ekaterina A. Akishina, Victor N. Khrustalev, Sixberth Mlowe

**Affiliations:** a Department of Organic Chemistry, Peoples’ Friendship University of Russia (RUDN University), 6 Miklukho-Maklaya St., 117198, Moscow, Russian Federation; b Department of Physics, Faculty of Sciences, Erciyes University, 38039 Kayseri, Turkey; c Laboratory of the Chemistry of Heterocyclic Compounds, Institute of Physical Organic Chemistry, National Academy of Sciences of Belarus, 13, Surganova Str., 220072, Minsk, Belarus; d N.D. Zelinsky Institute of Organic Chemistry, Russian Academy of Sciences, 47 Leninsky Av., Moscow, Russian Federation; e University of Dar es Salaam, Dar es Salaam University College of Education, Department of Chemistry, PO Box 2329, Dar es Salaam, Tanzania

**Keywords:** crystal structure, hydrogen bonds, C—H⋯π inter­actions, π–π stacking inter­actions, Hirshfeld surface analysis

## Abstract

The title compound, C_11_H_8_N_4_OS, crystallizes with two independent mol­ecules in the asymmetric unit. In the crystal, the N—H⋯N and C—H⋯N hydrogen bonds connect the mol­ecules, generating double layers parallel to the (001) plane. The layers are joined by C—H⋯π inter­actions to form a three-dimensional supra­molecular structure.

## Abstract 2

In the crystal, the mol­ecules are linked by N—H⋯N and C—H⋯N hydrogen bonds, forming double layers parallel to the (001) plane. The layers are connected by van der Waals inter­actions, generating a three-dimensional supra­molecular structure.

## Chemical context

Compounds with the five-membered isoxazole, iso­thia­zole and 1,3,4-thia­diazole heterocycles possess high potential for biological activity and are privileged scaffolds for the development of pharmaceutical agents (Das & Chanda, 2021 ▸; Kletskov *et al.*, 2020 ▸; Khalilullah *et al.*, 2014 ▸; Yadigarov *et al.*, 2009 ▸; Safavora *et al.*, 2019 ▸; Zubkov *et al.*, 2014 ▸). In particular, isoxazoles are able to enhance the action of ‘first-line’ anti­tumor substances, which makes it possible to reduce their therapeutic doses and thus reduce toxic side effects (Khalilov *et al.*, 2021 ▸; Kulchitsky *et al.*, 2012 ▸; Naghiyev *et al.*, 2020 ▸). The combination of the pharmacophore fragments of isoxazole and thia­diazole in one mol­ecule increases the variability of its binding to the key sites of enzymes regulating the biological action. The presence of an amino group additionally increases the biopotential of the mol­ecule, and the introduction of an aromatic fragment makes it possible to implement binding with a biotarget by π-stacking (Shixaliyev *et al.*, 2014 ▸, 2018 ▸; Mahmudov *et al.*, 2011 ▸, 2013 ▸; Gurbanov *et al.*, 2017 ▸, 2018*a* ▸,*b* ▸). To assess the biological potential of a mol­ecule *in silico* and the mol­ecular docking procedure, which is widely used for the development of new pharmaceuticals, information about the structures of promising mol­ecules is needed. All this initiated our research on the synthesis of 5-(5-phenyl­isoxazol-3-yl)-1,3,4-thia­diazol-2-amine (**1**) and the further determination of the accurate structure of its mol­ecule. The synthesis and structure of the compound has not published before. There are many approaches for building a thia­diazole heterocycle based on the use of carb­oxy­lic acids (Bhinge *et al.*, 2015 ▸; Nayak *et al.*, 2014 ▸), carbonyl chlorides (Sun *et al.*, 2001 ▸; Kudelko *et al.*, 2020 ▸), aldehydes (Shivakumara *et al.*, 2019 ▸; Wang *et al.*, 2019 ▸), *etc*. We chose here a method based on the transformation of carbo­nitriles (as shown in the scheme) as the shortest and most convenient way to achieve this purpose (Sakthivel *et al.*, 2016 ▸; *et al.*; Abdelhamid *et al.*, 2011 ▸). Its efficacy has recently been demonstrated by one of us (Petkevich *et al.*, 2021 ▸). The synthetic procedure involves the inter­action of 5-phenyl­isoxazole-3-carbo­nitrile with thio­semicarbazide. The starting 5-phenyl­isoxazole-3-carbo­nitrile was obtained according to the previously described method (Kulchitsky *et al.*, 2012 ▸; Bumagin *et al.*, 2018 ▸). 
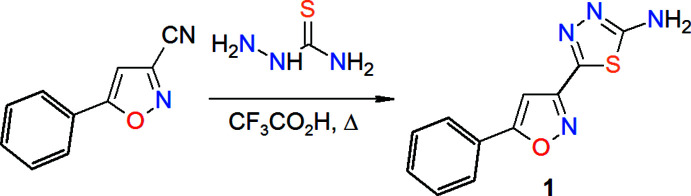


## Structural commentary

**Figure 1:**
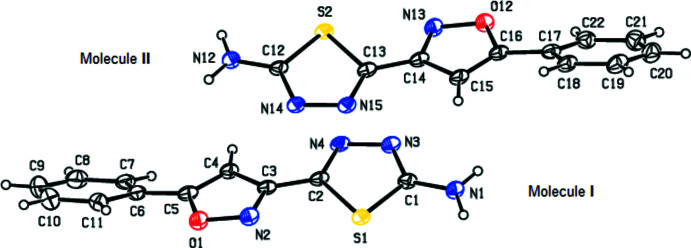
View of the two independent mol­ecules, I and II, in the asymmetric unit of the title compound, with displacement ellipsoids for the non-hydrogen atoms drawn at the 30% probability level.

**Figure 2:**
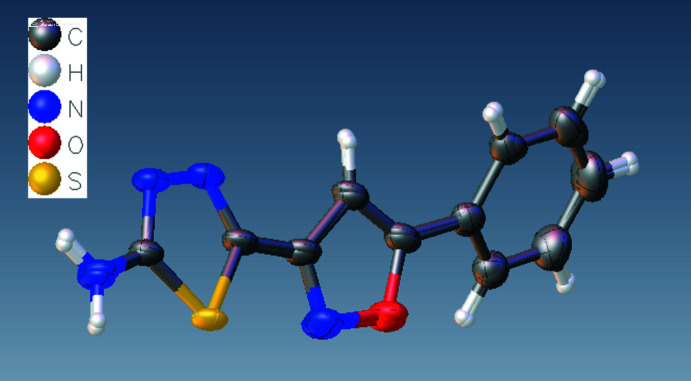
Overlay image of two independent mol­ecules in the asymmetric unit of the title compound.

The title compound **1** crystallizes in the ortho­rhom­bic space group *P*ca2_1_, with two independent mol­ecules (I with S1 and II with S2) in the asymmetric unit (Fig. 1 ▸). The oxazole (O1/N2/C3/C4/C5 and O12/N13/C14/C15/C16) and thia­diazole (S1/N3/N4/C1/C2 and S2/N14/N15/C12/C13) rings are essentially planar and inclined to one another by 18.8 (3) and 14.6 (3)° in mol­ecules I and II, respectively. The phenyl rings (C6–C11 and C17–C22) make dihedral angles of 24.6 (3) and 26.8 (3)° with the oxazole rings in mol­ecules I and II, respectively. Fig. 2 ▸ shows the overlay of mol­ecules I and II in the asymmetric unit, with an r.m.s. deviation of 0.087 Å. The C—N bond distances to the amino N atom of 1.330 (6) and 1.328 (6) Å, respectively, in mol­ecules I and II indicate strong conjugation of the amino groups with the thia­diazole π-systems.

## Supra­molecular features

**Figure 3:**
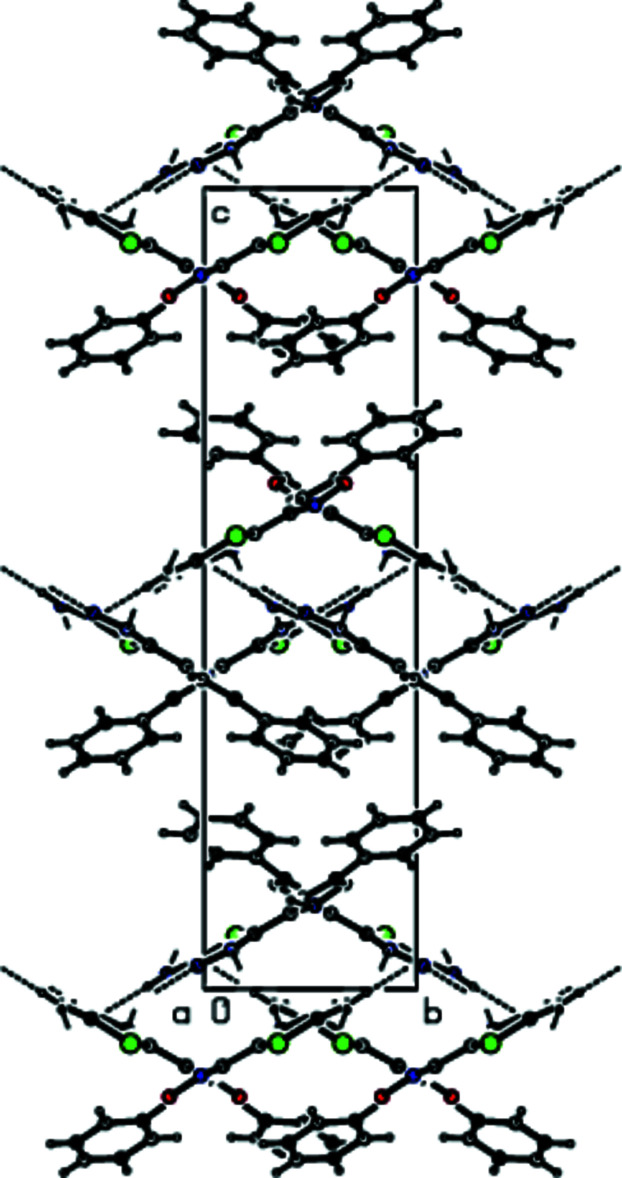
A view of the inter­molecular N—H⋯N and C—H⋯N inter­actions in the crystal structure of the title compound projected along the *a* axis.

**Figure 5:**
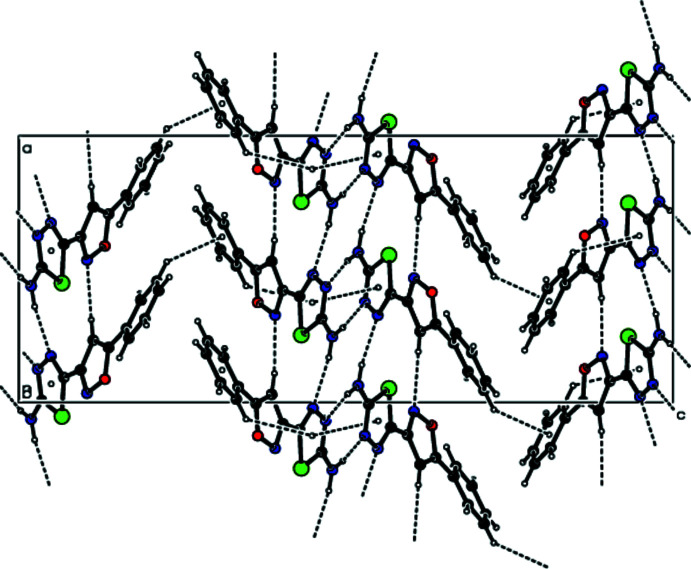
A view of the layer structure formed by inter­molecular N—H⋯N, C—H⋯N, C—H⋯π and π–π inter­actions in the crystal structure of the title compound projected along the *b* axis.

In the crystal, mol­ecules are linked by N—H⋯N and C—H⋯N hydrogen bonds (Table 1 ▸, Figs. 3 ▸ and 4 ▸), forming double layers of cross-linked mol­ecules parallel to the (001) plane. The mol­ecules within a layer are further linked by π–π stacking inter­actions between the thia­diazole rings [*Cg*1⋯*Cg*4(*x*, *y*, *z*) = 3.636 (3) Å, slippage = 1.283 Å, where *Cg*1 and *Cg*4 are the centroids of the rings S1/N3/N4/C1/C2 and S2/N14/N15/C12/C13, respectively]. The layers are linked by van der Waals inter­actions (Table 2 ▸), forming a three-dimensional supra­molecular structure (Fig. 5 ▸).

## Hirshfeld surface analysis

**Figure 6:**
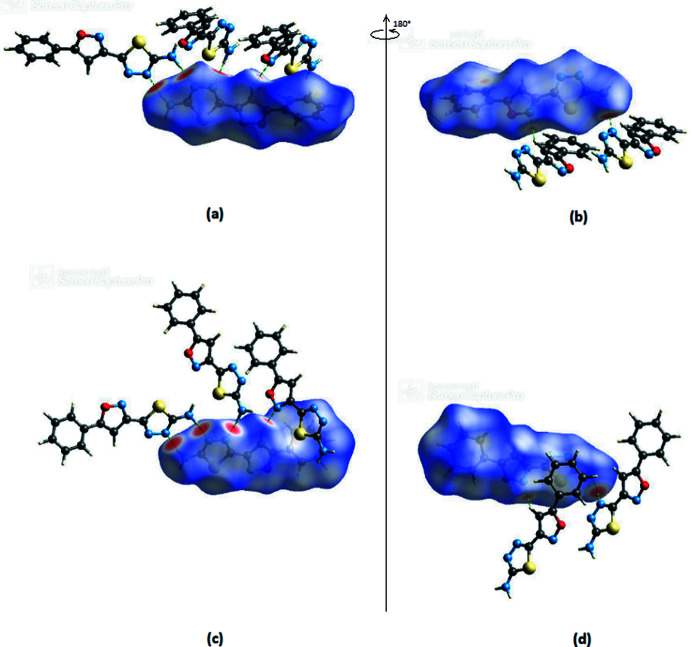
Front (*a*) and back (*b*) views of the three-dimensional Hirshfeld surface for mol­ecule I. Front (*c*) and back (*d*) views of the three-dimensional Hirshfeld surface for mol­ecule II. Some inter­molecular N—H⋯N and C—H⋯N inter­actions are shown as dashed lines.

*Crystal Explorer 17* (Turner *et al.*, 2017 ▸) was used to construct Hirshfeld surfaces for both independent mol­ecules in the asymmetric unit of the title compound. The *d*_norm_ mappings for mol­ecule I were performed in the range of −0.5418 to 1.2328 a.u., and for mol­ecule II in the range of −0.5446 to 1.1988 a.u. On the *d*_norm_ surfaces, bold red circles show the locations of N—H⋯N inter­actions. Smaller red spots are caused by C—H⋯N inter­actions (Fig. 6 ▸ *a*,*b* for mol­ecule I and Fig. 6 ▸ *c*,*d* for mol­ecule II).

**Figure 7:**
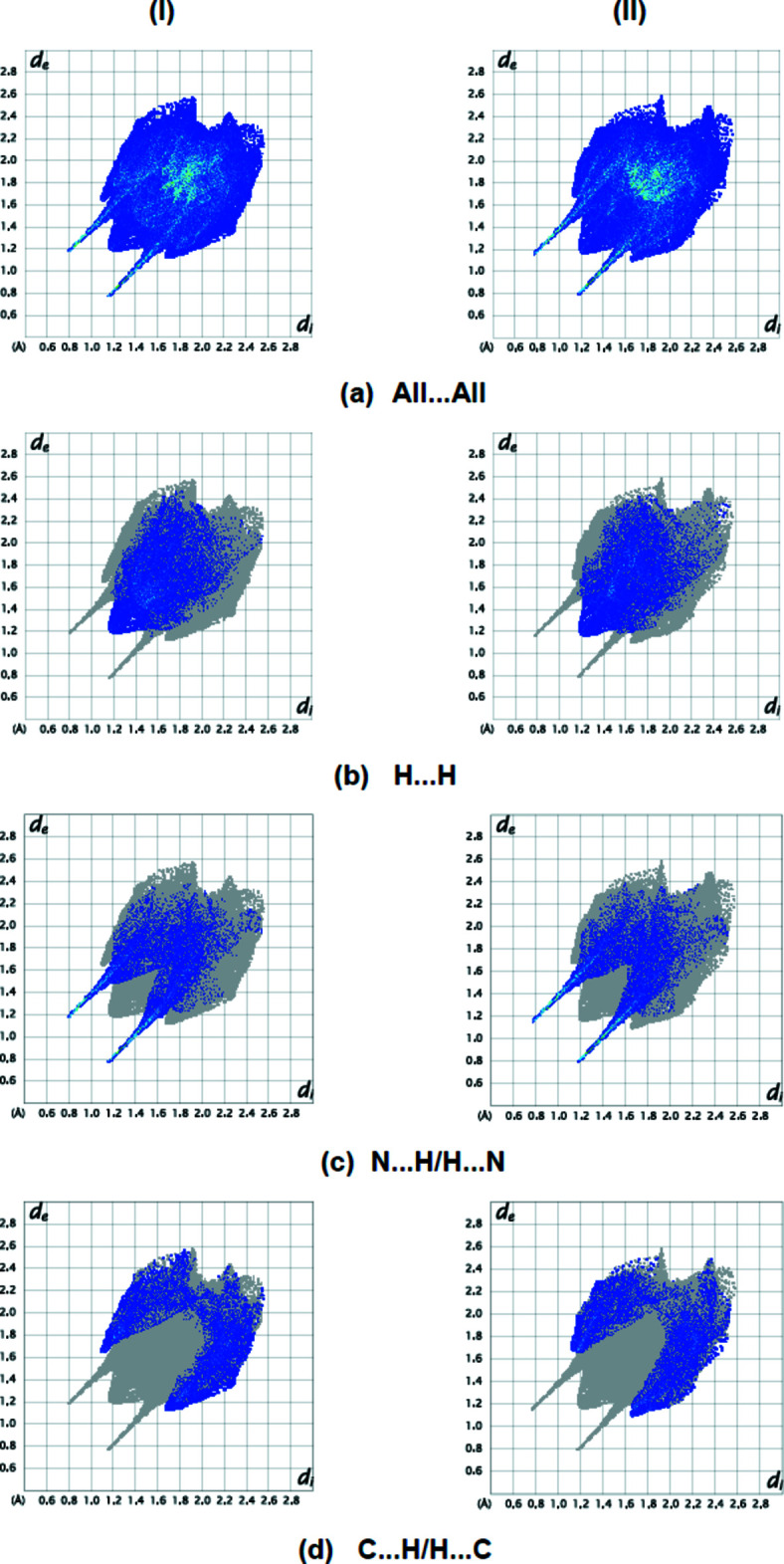
The two-dimensional fingerprint plots for mol­ecules I and II of the title compound showing (*a*) all inter­actions, and delineated into (*b*) H⋯H, (*c*) N⋯H/H⋯N and (*d*) C⋯H/H⋯C inter­actions. The *d*_i_ and *d*_e_ values are the closest inter­nal and external distances (in Å) from given points on the Hirshfeld surface.

Fingerprint plots (Fig. 7 ▸) reveal that while H⋯H (26.6% for mol­ecule I and 25.3% for mol­ecule II) inter­actions make the largest contributions to the surface contacts (Table 2 ▸), N⋯H/H⋯N (24.1% for I and 24.1% for II) and C⋯H/H⋯C (19.3% for I and 21.0% for II) contacts are also significant. The contributions of other, less noteworthy contacts are listed in Table 3 ▸. The environments of mol­ecules I and II are quite similar, as indicated in Table 3 ▸.

## Database survey

The only hit related to the title compound found in a search of the Cambridge Structural Database (CSD, Version 5.42; May 2021; Groom *et al.*, 2016 ▸) was 1-{[3-(thio­phen-2-yl)-4,5-di­hydro-1,2-oxazol-5-yl]meth­yl}-1*H*-indole-2,3-dione (NAQQOO: Rayni *et al.*, 2017 ▸). In the structure of NAQQOO, the indole ring system is almost planar as expected. The dihedral angle between this plane and that of the thio­phene ring is 2.01 (2)°. The mean plane of the isoxazole ring is inclined by 19.78 (14) and 20.83 (12)° to the thio­phene and indoline mean planes, respectively. In the crystal, the combin­ation of C—H⋯O hydrogen bonds forms stepped layers two mol­ecules thick, or slabs, which are oriented parallel to ( 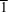 03). These layers are associated through offset π-stacking inter­actions, involving inversion-related indole rings in adjacent layers [inter­planar distance of 3.479 (1) Å], forming a supra­molecular three-dimensional structure.

## Synthesis and crystallization

**5-(5-Phenyl­isoxazol-3-yl)-1,3,4-thia­diazol-2-amine**:

Thio­semicarbazide (1.0 g, 11 mmol) was added at r.t to a solution of 5-phenyl­isoxazole-3-carbo­nitrile (1.70 g, 10 mmol) in CF_3_CO_2_H (10 mL), and the resulting mixture was heated under reflux for 6 h. After cooling, the mixture was poured into water (150 mL) and basified with 25% aqueous ammonia to pH ∼8. The precipitate was filtered off, washed with warm H_2_O (3 × 30 mL) and dried under reduced pressure over P_2_O_5_. The obtained solid product was recrystallized from MeOH giving light-yellow cubic crystals, yield 2.37 g (97%), m.p. = 501–503 K. IR (KBr), ν (cm^−1^): 3413, 3278, 3147, 3125, 2927, 1615, 1592, 1575, 1508, 1450, 1436, 1417, 1323, 1220, 1140, 1068, 947, 931, 817, 763, 686, 661, 629, 575. ^1^H NMR (DMSO-*d*_6_, 500 MHz, 301 K): δ = 7.51–7.58 (*m*, 4H, 3HAr + 1H-isox), 7.80 (*br.s*, 2H, NH_2_), 7.92–7.98 (*m*, 2HAr). ^13^C NMR (DMSO-*d*_6_, 125 MHz, 301 K): δ = 98.53 (CH-isox), 126.45 (2CHAr), 129.89 (2CHAr), 131.47 (1CHAr), 126.90, 145.57, 157.67, 170.46, 170.76 (5C). Mass-spectrum, *m*/*z* (*I*_rel_, %): 267 [*M*+Na]^+^ (5), 245 [*M*+H]^+^ (100). Elemental analysis calculated for C_11_H_8_N_4_OS (%): C 54.09, H 3.30, N 22.94, S 13.12; found (%): C 54.21, H 3.11, N 22.99, S 13.18.

## Refinement

Crystal data, data collection and structure refinement details are summarized in Table 4 ▸. All H atoms were positioned geometrically (N—H = 0.88 Å, C—H = 0.95 Å) and refined using a riding model with *U*_iso_(H) = 1.2*U*_eq_(N, C).

## Supplementary Material

Crystal structure: contains datablock(s) I. DOI: 10.1107/S2056989022003450/yk2168sup1.cif

Structure factors: contains datablock(s) I. DOI: 10.1107/S2056989022003450/yk2168Isup2.hkl

Supporting information file. DOI: 10.1107/S2056989022003450/yk2168Isup3.cml

CCDC reference: 2162503

Additional supporting information:  crystallographic information; 3D view; checkCIF report

## Figures and Tables

**Figure 4:**
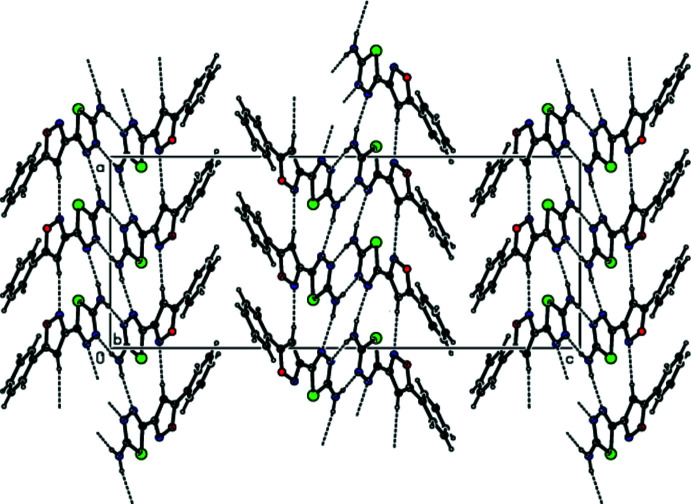
A view of the inter­molecular N—H⋯N and C—H⋯N inter­actions in the crystal structure of the title compound projected along the *b* axis.

**Table 1:** Hydrogen-bond geometry (Å, °) *Cg*4 and *Cg*6 are the centroids of the S2/N14/N15/C12/C13 and C17–C22 rings, respectively

*D*—H⋯*A*	*D*—H	H⋯*A*	*D*⋯*A*	*D*—H⋯*A*
N1—H1*A*⋯N14^i^	0.88	2.10	2.974 (6)	172
N1—H1*B*⋯N4^ii^	0.88	2.20	3.071 (5)	169
N12—H12*A*⋯N3^iii^	0.88	2.06	2.933 (6)	174
N12—H12*B*⋯N15^iv^	0.88	2.24	3.108 (5)	170
C4—H4⋯N2^iv^	0.95	2.56	3.363 (6)	142
C15—H15⋯N13^ii^	0.95	2.46	3.323 (6)	151
C8—H8⋯*Cg*6^v^	0.95	2.98	3.774 (6)	142
C22—H22⋯*Cg*4^i^	0.95	2.95	3.648 (6)	132

Symmetry codes: (i) 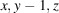 ; (ii) 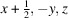 ; (iii) 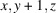 ; (iv) 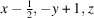 ; (v) 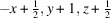 .

**Table 2:** Summary of short inter­atomic contacts (Å) in the title compound

Contact	Distance	Symmetry operation
S1⋯H12*B*	3.10	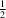 + *x*, 1 − *y*, *z*
N2⋯H4	2.56	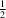 + *x*, 1 − *y*, *z*
N3⋯H12*A*	2.06	*x*, −1 + *y*, *z*
H1*B*⋯S2	3.09	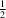 + *x*, −*y*, *z*
H1*B*⋯N4	2.20	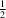 + *x*, −*y*, *z*
C2⋯N14	3.437 (7)	*x*, *y*, *z*
C4⋯H10	3.05	*x*, −1 + *y*, *z*
C7⋯H20	2.91	1 − *x*, −*y*, 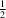 + *z*
H10⋯H19	2.49	1 − *x*, 1 − *y*, 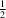 + *z*
H8⋯C18	2.87	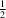 − *x*, 1 + *y*, 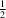 + *z*
H9⋯H21	2.59	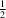 − *x*, 2 + *y*, 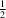 + *z*
N13⋯H15	2.46	− 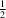 + *x*, −*y*, *z*
H12*B*⋯N15	2.24	− 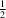 + *x*, 1 − *y*, *z*
C13⋯H22	2.91	*x*, 1 + *y*, *z*
C19⋯H22	2.94	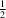 + *x*, −1 − *y*, *z*

**Table 3:** Percentage contributions of inter­atomic contacts to the Hirshfeld surface for the title compound

Contact	mol­ecule I	mol­ecule II
H⋯H	26.6	25.3
N⋯H/H⋯N	24.1	24.1
C⋯H/H⋯C	19.3	21.0
S⋯C/C⋯S	6.7	5.5
O⋯H/H⋯O	6.0	5.5
S⋯H/H⋯S	5.9	6.9
N⋯C/C⋯N	4.5	5.3
O⋯C/C⋯O	2.5	2.6
C⋯C	1.3	0.9
O⋯N/N⋯O	1.1	1.0
N⋯N	1.0	0.9
S⋯N/N⋯S	0.9	0.8
S⋯O/O⋯S	0.1	0.1

**Table 4:** Experimental details

Crystal data	
Chemical formula	C_11_H_8_N_4_OS
*M*_r_	244.27
Crystal system, space group	Orthorhombic, *P* *c* *a*2_1_
Temperature (K)	100
*a*, *b*, *c* (Å)	11.142 (2), 7.2555 (15), 27.333 (6)
*V* (Å^3^)	2209.6 (8)
*Z*	8
Radiation type	Mo *K*α
μ (mm^−1^)	0.28
Crystal size (mm)	0.24 × 0.18 × 0.02

Data collection	
Diffractometer	Bruker D8 QUEST PHOTON-III CCD
Absorption correction	Multi-scan (*SADABS*; Krause *et al.*, 2015 ▸)
*T*_min_, *T*_max_	0.924, 0.985
No. of measured, independent and observed [*I* > 2σ(*I*)] reflections	37296, 6442, 4347
*R*_int_	0.110
(sin θ/λ)_max_ (Å^−1^)	0.703

Refinement	
*R*[*F* ^2^ > 2σ(*F* ^2^)], *wR*(*F* ^2^), *S*	0.053, 0.125, 1.03
No. of reflections	6442
No. of parameters	307
No. of restraints	1
H-atom treatment	H-atom parameters constrained
Δρ_max_, Δρ_min_ (e Å^−3^)	0.32, −0.34
Absolute structure	Flack *x* determined using 1699 quotients [(*I* ^+^)−(*I* ^−^)]/[(*I* ^+^)+(*I* ^−^)] (Parsons et al., 2013 ▸)
Absolute structure parameter	0.44 (7)

Computer programs: *APEX3* (Bruker, 2018 ▸), *SAINT* (Bruker, 2013 ▸), *SHELXT* (Sheldrick, 2015*a* ▸), *SHELXL* (Sheldrick, 2015*b* ▸), *ORTEP-3 for Windows* (Farrugia, 2012 ▸) and *PLATON* (Spek, 2020 ▸).

## supplementary crystallographic information

### Crystal data

**Table:**

C_11_H_8_N_4_OS	*D*_x_ = 1.469 Mg m^−^^3^
*M**_r_* = 244.27	Mo *K*α radiation, λ = 0.71073 Å
Orthorhombic, *P**c**a*2_1_	Cell parameters from 5781 reflections
*a* = 11.142 (2) Å	θ = 2.8–28.1°
*b* = 7.2555 (15) Å	µ = 0.28 mm^−^^1^
*c* = 27.333 (6) Å	*T* = 100 K
*V* = 2209.6 (8) Å^3^	Plate, yellow
*Z* = 8	0.24 × 0.18 × 0.02 mm
*F*(000) = 1008	

### Data collection

**Table:**

Bruker D8 QUEST PHOTON-III CCD diffractometer	4347 reflections with *I* > 2σ(*I*)
φ and ω scans	*R*_int_ = 0.110
Absorption correction: multi-scan (SADABS; Krause *et al.*, 2015)	θ_max_ = 30.0°, θ_min_ = 2.8°
*T*_min_ = 0.924, *T*_max_ = 0.985	*h* = −15→15
37296 measured reflections	*k* = −10→10
6442 independent reflections	*l* = −38→38

### Refinement

**Table:**

Refinement on *F*^2^	Secondary atom site location: difference Fourier map
Least-squares matrix: full	Hydrogen site location: inferred from neighbouring sites
*R*[*F*^2^ > 2σ(*F*^2^)] = 0.053	H-atom parameters constrained
*wR*(*F*^2^) = 0.125	*w* = 1/[σ^2^(*F*_o_^2^) + 1.1115*P*] where *P* = (*F*_o_^2^ + 2*F*_c_^2^)/3
*S* = 1.03	(Δ/σ)_max_ < 0.001
6442 reflections	Δρ_max_ = 0.32 e Å^−^^3^
307 parameters	Δρ_min_ = −0.34 e Å^−^^3^
1 restraint	Absolute structure: Flack *x* determined using 1699 quotients [(*I*^+^)-(*I*^-^)]/[(*I*^+^)+(*I*^-^)] (Parsons et al., 2013)
Primary atom site location: difference Fourier map	Absolute structure parameter: 0.44 (7)

### Special details

**Table:**

Geometry. All esds (except the esd in the dihedral angle between two l.s. planes) are estimated using the full covariance matrix. The cell esds are taken into account individually in the estimation of esds in distances, angles and torsion angles; correlations between esds in cell parameters are only used when they are defined by crystal symmetry. An approximate (isotropic) treatment of cell esds is used for estimating esds involving l.s. planes.

### Fractional atomic coordinates and isotropic or equivalent isotropic displacement parameters (Å^2^)

**Table:**

	*x*	*y*	*z*	*U*_iso_*/*U*_eq_	
S1	0.55238 (9)	0.15104 (17)	0.56580 (4)	0.0346 (3)	
O1	0.4133 (3)	0.6622 (5)	0.63143 (14)	0.0407 (8)	
N1	0.5629 (3)	−0.1735 (6)	0.51880 (17)	0.0411 (11)	
H1A	0.5313	−0.2668	0.5028	0.049*	
H1B	0.6408	−0.1700	0.5241	0.049*	
N2	0.4659 (3)	0.5198 (6)	0.60417 (16)	0.0404 (10)	
N3	0.3753 (3)	−0.0298 (6)	0.52886 (16)	0.0368 (10)	
N4	0.3268 (3)	0.1278 (6)	0.54882 (15)	0.0363 (9)	
C1	0.4931 (4)	−0.0378 (7)	0.53495 (18)	0.0337 (11)	
C2	0.4066 (3)	0.2358 (7)	0.56877 (18)	0.0324 (10)	
C3	0.3784 (4)	0.4041 (7)	0.59427 (18)	0.0325 (10)	
C4	0.2667 (4)	0.4653 (7)	0.61365 (18)	0.0339 (10)	
H4	0.1905	0.4077	0.6107	0.041*	
C5	0.2934 (4)	0.6234 (7)	0.63716 (19)	0.0353 (11)	
C6	0.2228 (4)	0.7554 (7)	0.66463 (18)	0.0381 (11)	
C7	0.1161 (4)	0.6999 (7)	0.68654 (19)	0.0400 (11)	
H7	0.0917	0.5746	0.6847	0.048*	
C8	0.0454 (5)	0.8268 (9)	0.7110 (2)	0.0547 (16)	
H8	−0.0272	0.7883	0.7261	0.066*	
C9	0.0801 (6)	1.0089 (10)	0.7136 (2)	0.0622 (17)	
H9	0.0305	1.0958	0.7299	0.075*	
C10	0.1865 (6)	1.0655 (9)	0.6926 (2)	0.0586 (16)	
H10	0.2103	1.1910	0.6949	0.070*	
C11	0.2591 (5)	0.9399 (7)	0.66806 (19)	0.0435 (12)	
H11	0.3325	0.9788	0.6538	0.052*	
S2	0.25267 (9)	0.34769 (16)	0.43188 (5)	0.0345 (3)	
O12	0.3748 (3)	−0.1645 (5)	0.36528 (14)	0.0414 (8)	
N12	0.2466 (3)	0.6713 (6)	0.47918 (16)	0.0401 (10)	
H12A	0.2801	0.7631	0.4952	0.048*	
H12B	0.1686	0.6711	0.4740	0.048*	
N13	0.3275 (3)	−0.0167 (6)	0.39175 (17)	0.0406 (10)	
N14	0.4322 (3)	0.5220 (6)	0.46910 (15)	0.0354 (9)	
N15	0.4782 (3)	0.3622 (5)	0.44954 (15)	0.0336 (9)	
C12	0.3137 (4)	0.5330 (7)	0.46289 (18)	0.0325 (11)	
C13	0.3977 (3)	0.2571 (7)	0.42917 (19)	0.0324 (10)	
C14	0.4201 (4)	0.0858 (7)	0.40348 (19)	0.0339 (11)	
C15	0.5296 (4)	0.0103 (7)	0.38637 (18)	0.0355 (11)	
H15	0.6085	0.0576	0.3907	0.043*	
C16	0.4959 (4)	−0.1445 (7)	0.36254 (19)	0.0348 (10)	
C17	0.5587 (4)	−0.2878 (7)	0.33530 (18)	0.0354 (11)	
C18	0.6693 (4)	−0.2494 (8)	0.31302 (18)	0.0403 (12)	
H18	0.7024	−0.1289	0.3145	0.048*	
C19	0.7299 (5)	−0.3898 (8)	0.2886 (2)	0.0487 (14)	
H19	0.8046	−0.3646	0.2733	0.058*	
C20	0.6826 (5)	−0.5652 (9)	0.2866 (2)	0.0519 (14)	
H20	0.7252	−0.6607	0.2704	0.062*	
C21	0.5732 (5)	−0.6019 (8)	0.3082 (2)	0.0492 (14)	
H21	0.5400	−0.7223	0.3063	0.059*	
C22	0.5121 (5)	−0.4653 (7)	0.3324 (2)	0.0422 (12)	
H22	0.4372	−0.4923	0.3474	0.051*	

### Atomic displacement parameters (Å^2^)

**Table:**

	*U*^11^	*U*^22^	*U*^33^	*U*^12^	*U*^13^	*U*^23^
S1	0.0126 (4)	0.0464 (6)	0.0447 (7)	−0.0005 (4)	−0.0026 (4)	−0.0051 (6)
O1	0.0207 (14)	0.047 (2)	0.055 (2)	−0.0023 (14)	0.0010 (14)	−0.0061 (18)
N1	0.0154 (16)	0.047 (3)	0.061 (3)	0.0009 (17)	−0.0043 (17)	−0.011 (2)
N2	0.0187 (18)	0.049 (3)	0.053 (3)	−0.0012 (17)	0.0002 (17)	−0.006 (2)
N3	0.0159 (17)	0.047 (2)	0.048 (3)	−0.0013 (16)	−0.0018 (16)	−0.002 (2)
N4	0.0162 (17)	0.050 (3)	0.043 (2)	0.0009 (17)	−0.0030 (15)	0.0001 (19)
C1	0.0166 (19)	0.047 (3)	0.037 (3)	−0.0025 (18)	0.0005 (18)	0.000 (2)
C2	0.0130 (16)	0.048 (3)	0.036 (3)	0.0006 (17)	−0.0018 (18)	0.003 (2)
C3	0.0170 (19)	0.044 (3)	0.036 (3)	−0.0025 (18)	−0.0032 (17)	0.003 (2)
C4	0.0175 (19)	0.045 (3)	0.039 (3)	−0.0010 (19)	−0.0010 (18)	0.001 (2)
C5	0.0191 (19)	0.050 (3)	0.037 (3)	−0.0005 (18)	−0.0018 (19)	0.006 (2)
C6	0.031 (2)	0.049 (3)	0.034 (3)	0.006 (2)	−0.0073 (19)	0.000 (2)
C7	0.028 (2)	0.059 (3)	0.033 (3)	0.008 (2)	−0.005 (2)	−0.001 (2)
C8	0.038 (3)	0.086 (5)	0.040 (3)	0.012 (3)	0.002 (2)	−0.004 (3)
C9	0.062 (4)	0.071 (4)	0.053 (4)	0.020 (3)	0.003 (3)	−0.021 (3)
C10	0.064 (4)	0.056 (4)	0.056 (4)	0.005 (3)	−0.002 (3)	−0.012 (3)
C11	0.044 (3)	0.048 (3)	0.039 (3)	0.003 (2)	−0.003 (2)	−0.003 (2)
S2	0.0129 (4)	0.0473 (6)	0.0433 (6)	−0.0003 (5)	−0.0025 (4)	−0.0049 (6)
O12	0.0193 (15)	0.049 (2)	0.056 (2)	−0.0022 (14)	−0.0014 (15)	−0.0103 (18)
N12	0.0158 (17)	0.051 (3)	0.053 (3)	0.0013 (17)	−0.0033 (16)	−0.013 (2)
N13	0.0206 (19)	0.048 (3)	0.054 (3)	0.0026 (18)	−0.0004 (18)	−0.007 (2)
N14	0.0158 (17)	0.047 (2)	0.044 (2)	−0.0001 (15)	−0.0025 (15)	−0.0051 (19)
N15	0.0155 (16)	0.044 (2)	0.041 (2)	−0.0003 (15)	−0.0010 (14)	−0.0016 (18)
C12	0.0150 (19)	0.043 (3)	0.039 (3)	−0.0015 (18)	−0.0035 (17)	0.000 (2)
C13	0.0147 (16)	0.044 (3)	0.039 (3)	−0.0014 (17)	−0.0005 (18)	0.005 (2)
C14	0.0138 (18)	0.046 (3)	0.041 (3)	0.0009 (18)	−0.0021 (17)	0.004 (2)
C15	0.0135 (18)	0.051 (3)	0.042 (3)	0.0038 (19)	−0.0004 (18)	0.002 (2)
C16	0.0163 (19)	0.051 (3)	0.037 (2)	0.0032 (19)	−0.0007 (17)	0.005 (2)
C17	0.025 (2)	0.048 (3)	0.033 (3)	0.003 (2)	−0.0036 (19)	0.004 (2)
C18	0.021 (2)	0.056 (3)	0.044 (3)	0.005 (2)	−0.0004 (18)	0.004 (2)
C19	0.033 (3)	0.070 (4)	0.042 (3)	0.014 (2)	0.008 (2)	0.009 (3)
C20	0.051 (3)	0.061 (4)	0.044 (3)	0.018 (3)	0.003 (3)	−0.002 (3)
C21	0.052 (4)	0.050 (3)	0.045 (3)	0.002 (3)	0.003 (3)	0.000 (3)
C22	0.035 (3)	0.052 (3)	0.039 (3)	0.001 (2)	−0.001 (2)	0.000 (2)

### Geometric parameters (Å, º)

**Table:**

S1—C1	1.739 (5)	S2—C12	1.729 (5)
S1—C2	1.739 (4)	S2—C13	1.746 (4)
O1—C5	1.374 (5)	O12—C16	1.359 (5)
O1—N2	1.402 (5)	O12—N13	1.398 (5)
N1—C1	1.330 (6)	N12—C12	1.328 (6)
N1—H1A	0.8800	N12—H12A	0.8800
N1—H1B	0.8800	N12—H12B	0.8800
N2—C3	1.315 (6)	N13—C14	1.312 (6)
N3—C1	1.324 (5)	N14—C12	1.334 (5)
N3—N4	1.377 (6)	N14—N15	1.376 (5)
N4—C2	1.304 (6)	N15—C13	1.302 (6)
C2—C3	1.441 (7)	C13—C14	1.449 (7)
C3—C4	1.424 (6)	C14—C15	1.417 (6)
C4—C5	1.348 (7)	C15—C16	1.352 (7)
C4—H4	0.9500	C15—H15	0.9500
C5—C6	1.449 (7)	C16—C17	1.458 (7)
C6—C7	1.391 (7)	C17—C22	1.391 (7)
C6—C11	1.402 (7)	C17—C18	1.402 (7)
C7—C8	1.384 (7)	C18—C19	1.393 (7)
C7—H7	0.9500	C18—H18	0.9500
C8—C9	1.378 (9)	C19—C20	1.379 (8)
C8—H8	0.9500	C19—H19	0.9500
C9—C10	1.379 (9)	C20—C21	1.380 (8)
C9—H9	0.9500	C20—H20	0.9500
C10—C11	1.391 (8)	C21—C22	1.373 (7)
C10—H10	0.9500	C21—H21	0.9500
C11—H11	0.9500	C22—H22	0.9500
			
C1—S1—C2	86.9 (2)	C12—S2—C13	87.1 (2)
C5—O1—N2	108.4 (3)	C16—O12—N13	108.7 (4)
C1—N1—H1A	120.0	C12—N12—H12A	120.0
C1—N1—H1B	120.0	C12—N12—H12B	120.0
H1A—N1—H1B	120.0	H12A—N12—H12B	120.0
C3—N2—O1	105.7 (4)	C14—N13—O12	105.3 (4)
C1—N3—N4	112.0 (4)	C12—N14—N15	111.7 (4)
C2—N4—N3	113.4 (4)	C13—N15—N14	113.8 (4)
N3—C1—N1	124.7 (4)	N12—C12—N14	124.0 (4)
N3—C1—S1	113.8 (4)	N12—C12—S2	122.1 (3)
N1—C1—S1	121.5 (3)	N14—C12—S2	113.9 (4)
N4—C2—C3	124.3 (4)	N15—C13—C14	126.2 (4)
N4—C2—S1	113.9 (4)	N15—C13—S2	113.5 (4)
C3—C2—S1	121.7 (3)	C14—C13—S2	120.2 (3)
N2—C3—C4	111.9 (4)	N13—C14—C15	112.2 (5)
N2—C3—C2	118.6 (4)	N13—C14—C13	117.9 (4)
C4—C3—C2	129.4 (4)	C15—C14—C13	129.8 (4)
C5—C4—C3	104.4 (4)	C16—C15—C14	103.9 (4)
C5—C4—H4	127.8	C16—C15—H15	128.0
C3—C4—H4	127.8	C14—C15—H15	128.0
C4—C5—O1	109.6 (4)	C15—C16—O12	109.8 (4)
C4—C5—C6	133.6 (4)	C15—C16—C17	134.9 (4)
O1—C5—C6	116.8 (4)	O12—C16—C17	115.4 (4)
C7—C6—C11	119.6 (5)	C22—C17—C18	119.2 (5)
C7—C6—C5	119.7 (5)	C22—C17—C16	120.6 (5)
C11—C6—C5	120.6 (5)	C18—C17—C16	120.1 (5)
C8—C7—C6	120.1 (5)	C19—C18—C17	119.2 (5)
C8—C7—H7	119.9	C19—C18—H18	120.4
C6—C7—H7	119.9	C17—C18—H18	120.4
C9—C8—C7	120.2 (6)	C20—C19—C18	120.6 (5)
C9—C8—H8	119.9	C20—C19—H19	119.7
C7—C8—H8	119.9	C18—C19—H19	119.7
C8—C9—C10	120.4 (6)	C19—C20—C21	119.9 (5)
C8—C9—H9	119.8	C19—C20—H20	120.0
C10—C9—H9	119.8	C21—C20—H20	120.0
C9—C10—C11	120.3 (6)	C22—C21—C20	120.4 (6)
C9—C10—H10	119.8	C22—C21—H21	119.8
C11—C10—H10	119.8	C20—C21—H21	119.8
C10—C11—C6	119.4 (5)	C21—C22—C17	120.7 (5)
C10—C11—H11	120.3	C21—C22—H22	119.7
C6—C11—H11	120.3	C17—C22—H22	119.7
			
C5—O1—N2—C3	0.5 (5)	C16—O12—N13—C14	0.4 (5)
C1—N3—N4—C2	0.8 (6)	C12—N14—N15—C13	0.3 (6)
N4—N3—C1—N1	−179.2 (5)	N15—N14—C12—N12	178.6 (5)
N4—N3—C1—S1	−0.2 (5)	N15—N14—C12—S2	−0.7 (5)
C2—S1—C1—N3	−0.2 (4)	C13—S2—C12—N12	−178.6 (5)
C2—S1—C1—N1	178.7 (5)	C13—S2—C12—N14	0.7 (4)
N3—N4—C2—C3	−176.7 (5)	N14—N15—C13—C14	176.6 (5)
N3—N4—C2—S1	−1.0 (6)	N14—N15—C13—S2	0.3 (6)
C1—S1—C2—N4	0.7 (4)	C12—S2—C13—N15	−0.5 (4)
C1—S1—C2—C3	176.5 (4)	C12—S2—C13—C14	−177.1 (4)
O1—N2—C3—C4	0.7 (6)	O12—N13—C14—C15	−0.9 (6)
O1—N2—C3—C2	−176.3 (4)	O12—N13—C14—C13	176.6 (4)
N4—C2—C3—N2	−166.4 (5)	N15—C13—C14—N13	169.7 (5)
S1—C2—C3—N2	18.2 (7)	S2—C13—C14—N13	−14.2 (7)
N4—C2—C3—C4	17.1 (9)	N15—C13—C14—C15	−13.3 (9)
S1—C2—C3—C4	−158.3 (4)	S2—C13—C14—C15	162.9 (4)
N2—C3—C4—C5	−1.7 (6)	N13—C14—C15—C16	1.1 (6)
C2—C3—C4—C5	174.9 (5)	C13—C14—C15—C16	−176.1 (5)
C3—C4—C5—O1	2.0 (6)	C14—C15—C16—O12	−0.7 (6)
C3—C4—C5—C6	−180.0 (5)	C14—C15—C16—C17	178.6 (6)
N2—O1—C5—C4	−1.7 (6)	N13—O12—C16—C15	0.2 (6)
N2—O1—C5—C6	179.9 (4)	N13—O12—C16—C17	−179.2 (4)
C4—C5—C6—C7	24.7 (9)	C15—C16—C17—C22	152.5 (6)
O1—C5—C6—C7	−157.4 (4)	O12—C16—C17—C22	−28.2 (7)
C4—C5—C6—C11	−153.3 (6)	C15—C16—C17—C18	−25.4 (9)
O1—C5—C6—C11	24.5 (7)	O12—C16—C17—C18	154.0 (5)
C11—C6—C7—C8	0.9 (8)	C22—C17—C18—C19	−0.2 (7)
C5—C6—C7—C8	−177.2 (5)	C16—C17—C18—C19	177.7 (5)
C6—C7—C8—C9	0.3 (8)	C17—C18—C19—C20	−0.4 (8)
C7—C8—C9—C10	−1.2 (10)	C18—C19—C20—C21	1.0 (9)
C8—C9—C10—C11	0.8 (10)	C19—C20—C21—C22	−1.1 (9)
C9—C10—C11—C6	0.4 (9)	C20—C21—C22—C17	0.5 (9)
C7—C6—C11—C10	−1.2 (8)	C18—C17—C22—C21	0.1 (8)
C5—C6—C11—C10	176.8 (5)	C16—C17—C22—C21	−177.8 (5)

### Hydrogen-bond geometry (Å, º)

*Cg*4 and *Cg*6 are the centroids of the S2/N14/N15/C12/C13 and C17–C22 rings, respectively

**Table:**

*D*—H···*A*	*D*—H	H···*A*	*D*···*A*	*D*—H···*A*
N1—H1*A*···N14^i^	0.88	2.10	2.974 (6)	172
N1—H1*B*···N4^ii^	0.88	2.20	3.071 (5)	169
N12—H12*A*···N3^iii^	0.88	2.06	2.933 (6)	174
N12—H12*B*···N15^iv^	0.88	2.24	3.108 (5)	170
C4—H4···N2^iv^	0.95	2.56	3.363 (6)	142
C15—H15···N13^ii^	0.95	2.46	3.323 (6)	151
C8—H8···*Cg*6^v^	0.95	2.98	3.774 (6)	142
C22—H22···*Cg*4^i^	0.95	2.95	3.648 (6)	132

Symmetry codes: (i) *x*, *y*−1, *z*; (ii) *x*+1/2, −*y*, *z*; (iii) *x*, *y*+1, *z*; (iv) *x*−1/2, −*y*+1, *z*; (v) −*x*+1/2, *y*+1, *z*+1/2.
